# *In Vitro* Antimicrobial Activity of Plant-Derived Diterpenes against Bovine Mastitis Bacteria

**DOI:** 10.3390/molecules18077865

**Published:** 2013-07-04

**Authors:** Ariana P. Fonseca, Fernanda T. Estrela, Thaís S. Moraes, Luiza J. Carneiro, Jairo K. Bastos, Raquel A. dos Santos, Sérgio R. Ambrósio, Carlos H. G. Martins, Rodrigo C. S. Veneziani

**Affiliations:** 1 Nucleus of Research in Sciences and Technology, University of Franca, Armando Salles de Oliveira Road, 201, Franca 1404-600, Brazil; 2 Faculty of Pharmaceutical Sciences of Ribeirão Preto, Laboratory of Pharmacognosy, University of São Paulo, Café Avenue, Ribeirão Preto 14040-903, Brazil

**Keywords:** diterpenes, bovine mastitis, antibacterial, *ent-*copalic acid

## Abstract

We evaluated the antibacterial activity of three diterpenes isolated from natural sources against a panel of microorganisms responsible for bovine mastitis. *ent*-Copalic acid (CA) was the most active metabolite, with promising MIC values (from 1.56 to 6.25 µg mL^−1^) against *Staphylococcus aureus* (ATCC and clinical isolate), *Staphylococcus epidermidis*, *Streptococcus agalactiae*, and *Streptococcus dysgalactiae*. We conducted time-kill assays of CA against *S. aureus*, a commensal organism considered to be a ubiquitous etiological agent of bovine mastitis in dairy farms worldwide. In the first 12 h, CA only inhibited the growth of the inoculums (bacteriostatic effect), but its bactericidal effect was clearly noted thereafter (between 12 and 24 h). In conclusion, CA should be considered for the control of several Gram-positive bacteria related to bovine mastitis.

## 1. Introduction

Bovine mastitis is an inflammatory condition that affects the mammary glands of cows. It has a significant impact on animal production, animal welfare, and milk quality [1]. Several Gram-positive and Gram-negative bacteria such as *Staphylococcus aureus*, *Escherichia coli*, *Staphylococcus epidermidis*, *Streptococcus agalactiae*, and *Streptococcus dysgalactiae* are responsible for this pathology, considered one of the main causes of economic losses in the dairy industry [2,3]. The successful control of this condition generally relies on treatment with antibiotics [1] but this practice contributes to the widespread use of these medications in dairy farms, culminating in antibiotic-resistant bacterial strains [2,3]. Therefore, it is necessary to discover novel and safe compounds for the control of this disease.

Natural products are a rich and promising source for the discovery of new biologically active compounds [4]. Among plant metabolites, diterpenes display a wide spectrum of biological activities, including antibacterial action [5,6,7,8]. Besides, a search of the scientific literature using PubMed and SciFinder databases reveals that several classes of diterpenoids are considered potential sources of antimicrobial agents [9,10,11,12,13,14].

In recent years, our research group has investigated the antibacterial activity of naturally occurring diterpenes against bacterial strains responsible for human pathologies like caries, periodontitis, pneumonia, and nosocomial infections, among others. We observed that manool (MO), *ent-*kaurenoic acid (KA), and *ent-*copalic acid (CA) were the most promising compounds for most of the tested bacteria [15,16,17,18].

Considering the growing need for new antibacterial agents that can be used to control and treat bovine mastitis and as part of our ongoing efforts to explore the antibacterial properties of diterpenes, here we investigate the antimicrobial activity of MO, KA, and CA against a representative panel of bacteria responsible for this pathology.

## 2. Results and Discussion

**Figure 1:**
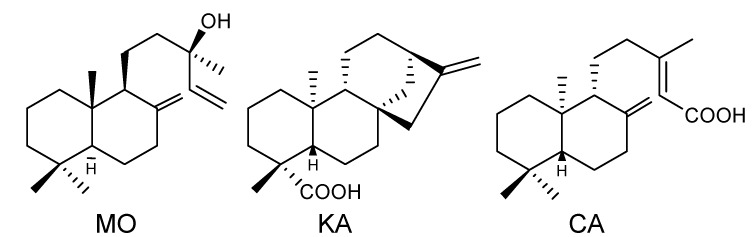
Chemical structures of the isolated diterpenes: manool (MO), *ent-*kaurenoic acid (KA), and *ent-*copalic acid (CA).

The isolation procedures described in the Experimental section (3.1. Compound Isolation and Identification) furnished the three diterpenes selected for this study (Figure 1). The compounds were identified by ^1^H and ^13^C-NMR, and by comparison with literature data as MO [19,20]; KA [21] and CA [22]. The NMR spectra also indicated that the purity of the isolated compounds lay between 95 and 98%.

The antimicrobial effects of MO, KA, and CA were evaluated against a panel of ATCC and clinically isolated bacteria associated with bovine mastitis. Table 1 lists the MIC values obtained for the metabolites.

**Table 1:** *In vitro* antibacterial activity (MIC) of manool (MO), *ent-*kaurenoic acid (KA), and *ent-*copalic acid (CA).

Bacteria	MIC in µg mL^−1^ (MIC in µM)			
	Positive control ^1^	MO	KA	CA ^2^
*S. aureus* (ATCC 6538)	0.05 (0.05)	*	*	6.25 (20.54)
*S. aureus* (clinical isolate)	>5.89 (>6.48)	200.00 (689.03)	*	6.25 (20.54)
*E. coli* (ATCC 14948)	>1.50 (>1.65)	*	*	*
*E. coli* (clinical isolate)	>2.95 (>3.25)	*	*	*
*S. epidermidis* (ATCC 12228)	0.05 (0.05)	100.00 (344.51)	25.00 (82.78)	6.25 (20.54)
*S. agalactiae* (ATCC 12386)	0.05 (0.05)	6.25 (21.53)	3.12 (10.33)	1.56 (5.13)
*S. dysgalactiae* (ATCC 12238)	0.05 (0.05)	6.25 (21.53)	3.12 (10.33)	1.56 (5.13)

* MIC values higher than 400.00 µg mL^−1^; ^1^ Penicillin was used as positive control. The only exception was *E. coli*, for which gentamicin was utilized; ^2^ MIC and MBC values for CA were the same for all the tested microorganisms.

Some authors have proposed criteria based on MIC values for the determination of the antimicrobial potential of compounds isolated from natural sources [23,24]. These authors suggest that MIC values higher than 100.0 µg mL^−1^ for pure metabolites are evidence of poor activity, while isolated compounds that inhibit the growth of microorganisms at concentrations below 10.0 µg mL^−1^ may be considered promising leads in the search for new anti-infective agents. Bearing these criteria in mind, analysis of the results presented in Table 1 showed that MO, KA, and CA were effective against *S. agalactiae* and *S. dysgalactiae*. CA was the most active compound, with promising MIC values for most of the investigated bacteria. None of the tested compounds was active against the tested Gram-negative bacterium (*E. coli*).

CA at 6.25 µg mL^−1^ completely inhibited this microorganism, suggesting that this diterpene is effective against different strains of this species. Moreover, CA was not cytotoxic to the human fibroblast cell line at concentrations up to 62.5 µM. All these results confirm the role of CA as an important and selective metabolite that should be considered for the control of *S. aureus*.

Gibbons has pointed out that diterpenes are one of the largest groups of plant-derived compounds with potential activity against *S. aureus*, a commensal organism that is considered to be a ubiquitous etiological agent of bovine mastitis in dairy farms worldwide [25,26]. Our findings reinforce the role of this class of metabolites against the main pathogen involved in this disease. However, despite the importance of diterpenes as a source for the discovery of new lead anti-infection compounds, reports on the antimicrobial activity of this class of natural products against pathogens responsible for bovine mastitis are scarce. This is the first time that isolated diterpenes have been tested against a panel of microorganisms associated with this infection. Therefore, the present study is important in the search for novel compounds that can be applied for the control of a disease with significant impact on animal production, animal welfare, and milk quality. 

**Figure 2:**
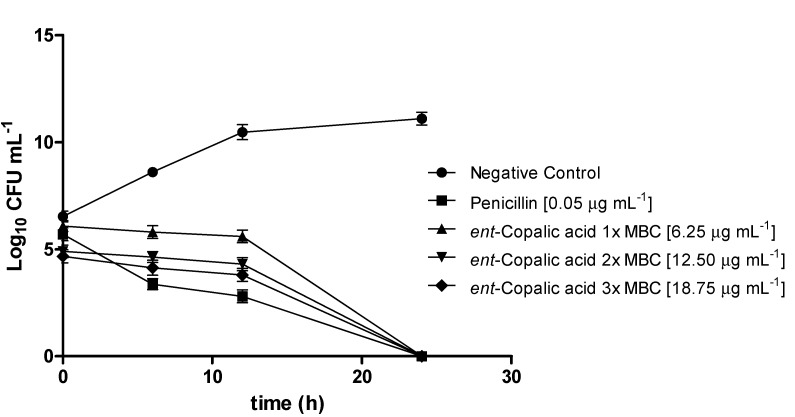
Time-kill curves for CA against *S. aureus*.

Because CA was active against *S. aureus*, one of the etiological agents of bovine mastitis, we decided to investigate other aspects of this antimicrobial activity. Hence, we conducted a time-kill curve set of experiments, to determine how long it is necessary for CA to completely eliminate this pathogen. Figure 2 depicts the obtained time-kill curves.

At its MBC (6.25 μg mL^−1^), CA is able to completely kill *S. aureus* after only 24 h of incubation. In the first 12 h, CA only inhibits the growth of the inoculum, indicating that this metabolite displays a bacteriostatic effect during this period, with its bactericidal effect being clearly noted thereafter (between 12 and 24 h). The periods of time investigated for each concentration were not statistically different, showing the lack of dose-dependent responses for this compound in the assayed conditions.

## 3. Experimental

### 3.1. Compound Isolation and Identification

KA and CA were isolated and identified from certified dried leaves of *Mikania hirsutissima* (purchased from Nutri Comércio de Ervas LTDA, São Paulo, SP, Brazil) and from authentic oleoresin of *Copaifera langsdorffii* (kindly provided by the Brazilian Company Apis-Flora Comercial e Industrial, Ribeirão Preto, SP, Brazil), respectively, after application of several chromatographic techniques and according to previously described methodologies [16,17,18]. To obtain MO, certified *Salvia officinalis* dried leaves (1.0 Kg) were purchased from Nutri Comércio de Ervas LTDA, pulverized, and exhaustively extracted for 48 h at room temperature with dichloromethane (5 L), to give 45.5 g of the crude extract, which was suspended in 300 mL methanol/H_2_O (9:1) and filtered. The soluble fraction was partitioned using *n-*hexane (300 mL, three times), to yield 10.6 g of hexane-soluble fraction after solvent evaporation under reduced pressure. This fraction was submitted to several chromatographic procedures (vacuum chromatography—VC, Thin layer chromatography—TLC, and High Pressure Liquid Chromatography—HPLC), to afford 200 mg of MO after ^1^H- and ^13^C-NMR analyses [19].

### 3.2. Bacterial Strains and Antimicrobial Testing

Most of the tested strains were obtained from the American Type Culture Collection (ATCC): *Staphylococcus aureus* (ATCC 6538), *Escherichia coli* (ATCC 14948), *Staphylococcus epidermidis*(ATCC 12228), *Streptococcus agalactiae* (ATCC 12836), and *Streptococcus dysgalactiae* (ATCC 12388). Dra. Maria Aparecida Vasconcelos Paiva Brito from “Embrapa Gado de Leite” (Juiz de Fora-MG) kindly provided the isolated strains from clinical bovine mastitis cases. All strains were kept in our laboratory and cryopreserved at −86 °C. They were maintained in the brain-heart infusion (BHI) broth containing 20% (v/v) glycerol. The minimal inhibitory concentration (MIC; lowest concentration of the compound able to inhibit microorganism growth) and the minimal bactericidal concentration (MBC; lowest concentration of the compound at which 99.99% or more of the initial inoculum was killed) were determined in triplicate, using the microdilution broth method in 96-well microplates. Samples were dissolved in dimethyl sulfoxide (DMSO; Synth, Diadema, SP, Brazil) at 1 mg mL^−1^ and diluted in tryptic soy broth (Difco, Sparks, MD, USA), which afforded concentrations ranging from 400.0 to 1.56 µg mL^−1^. The final DMSO content in the solutions was 5% (v/v). A 5% (v/v) DMSO solution was used as negative control. The inoculum was adjusted for each organism, to yield a cell concentration of 5 × 10^5^ colony forming units (CFU) per mL, according to previous standardization by the Clinical Laboratory Standards Institute [27]. One inoculated well was included, to control the adequacy of the broth for organism growth. One non-inoculated well, free of antimicrobial agent, was also employed, to ensure medium sterility. Penicillin was used as positive control. The only exception was *E. coli*, for which gentamicin was utilized as positive control. The microplates (96-wells) were sealed with plastic film and incubated at 37 °C for 24 h. Then, an aqueous resazurin solution (0.02%) was added to the microplates, to indicate microorganism viability. To determine MBC values, an aliquot of the inoculum was aseptically removed from each well with no apparent bacterial growth before addition of resazurin. The aliquot was then plated onto tryptic soy agar supplemented with 5% sheep blood. The plates were incubated as previously described [27]. MBC values were only determined for the most active compound (CA).

### 3.3. Kill Kinetics

Time-kill assays were performed in triplicate based on D’Arrigo *et al.* [28]. Only CA against *Staphylococcus aureus* (ATCC 6538) was tested, because this compound displayed the highest antimicrobial activity. Tubes containing CA at final concentrations of 6.25, 12.5, and 18.7 µg mL^−1^ (respectively one, two, and three-times the MBC value of CA for *S. aureus*) were inoculated with the tested microorganism at a starting bacterial density of 5 × 10^5^ CFU mL^−1^ and incubated at 37 °C. At 6, 12, and 24 h after incubation, samples were removed and diluted in sterile fresh medium, when necessary, for determination of viable strains. The diluted samples (50 µL) were spread onto tryptic soy agar, incubated at 37 °C, and counted after 48 h. Time-kill curves were constructed by plotting the log10 CFU mL^−1^ versus time. The assays were performed in triplicate for each concentration as well as the positive (penicillin at its MBC, PEN, 0.046 µg mL^−1^) and negative controls (suspension of *S. aureus* without added CA).

### 3.4. Cytotoxicity Assay

The effect of CA on cell viability was assessed by the XTT assay using the primary human fibroblast cell line obtained from Coriell Cell Repositories (Camden, NJ, USA). Briefly, cells were trypsinized and seeded in 96-well plate at a concentration of 10^4^ cells/well in DMEM plus HAM-F10 (1:1, v/v) medium (Sigma, St. Louis, MO, USA) supplemented with 20% fetal bovine serum (Life Technologies, CA, USA). After 24 h of incubation at 37 °C, cell cultures were treated with different concentrations of CA dissolved in DMSO (1%), namely from 7.8 to 62.5 uM, for further 24 h. Cell viability was assessed with the Cell Proliferation Kit II (Roche, Mannheim, Germany) according to the manufacturer’s instruction. Absorbance of the orange formazan product was detected at 490 nm, reference wavelength at 620 nm, in a microplate reader Sunrise (Tecam, Männdorf, Switzerland). Cell viability was expressed as the percentage of negative control. Doxorubicin at 3.0 µg mL^−1^ was used as positive control.

## 4. Conclusions

*ent-*Copalic acid is an important diterpene for the control of several Gram-positive bacteria related to bovine mastitis. The results reinforce the relevance of this class of natural products as a source of new potential antimicrobial compounds for the control of several human and animal infectious diseases.
